# Adipocyte Secreted Factors Enhance Aggressiveness of Prostate Carcinoma Cells

**DOI:** 10.1371/journal.pone.0123217

**Published:** 2015-04-30

**Authors:** Ângela Moreira, Sofia S. Pereira, Madalena Costa, Tiago Morais, Ana Pinto, Rúben Fernandes, Mariana P. Monteiro

**Affiliations:** 1 Department of Anatomy, Unit for Multidisciplinary Research in Biomedicine (UMIB), Institute for Biomedical Sciences Abel Salazar (ICBAS), University of Porto, Porto, Portugal; 2 Ciências Químicas e das Biomoléculas (CQB), Escola Superior de Tecnologia da Saúde do Porto do Instituto Politécnico do Porto (ESTSP-IPP), Vila Nova de Gaia, Portugal; 3 Centro de Investigação em Saúde e Ambiente (CISA), Escola Superior de Tecnologia da Saúde do Porto do Instituto Politécnico do Porto (ESTSP-IPP), Vila Nova de Gaia, Portugal; Innsbruck Medical University, AUSTRIA

## Abstract

Obesity has been associated with increased incidence and risk of mortality of prostate cancer. One of the proposed mechanisms underlying this risk association is the change in adipokines expression that could promote the development and progression of the prostate tumor cells. The main goal of this study was to evaluate the effect of preadipocyte and adipocyte secretome in the proliferation, migration and invasion of androgen independent prostate carcinoma cells (RM1) and to assess cell proliferation in the presence of the adiposity signals leptin and insulin. RM1 cells were co-cultured in with preadipocytes, adipocytes or cultured in their respective conditioned medium. Cell proliferation was assessed by flow cytometry and XTT viability test. Cell migration was evaluated using a wound healing injury assay of RM1 cells cultured with conditioned media. Cellular invasion of RM1 cells co-cultured with adipocytes and preadipocytes was assessed using matrigel membranes. Preadipocyte conditioned medium was associated with a small increase in RM1 proliferation, while adipocytes conditioned media significantly increased RM1 cell proliferation (p<0.01). Adipocytes also significantly increased the RM1 cells proliferation in co-culture (p <0.01). Cell migration was higher in RM1 cells cultured with preadipocyte and adipocyte conditioned medium. RM1 cell invasion was significantly increased after co-culture with preadipocytes and adipocytes (p <0.05). Insulin also increased significantly the cell proliferation in contrast to leptin, which showed no effect. In conclusion, prostate carcinoma cells seem to be influenced by factors secreted by adipocytes that are able to increase their ability to proliferate, migrate and invade.

## Introduction

Prostate cancer is the second most common cancer in men, the sixth leading cause of cancer-related death worldwide [1], and the most common cancer in males, in Europe [2].

Prostate cancer is often an indolent disease, however 30% of the tumors become aggressive [3]. Typically, prostate cancer cells are initially androgen dependent, but can become androgen-independent with the progression of the disease to a more advanced and aggressive stage, leading to a worse prognosis [3].

Several risk factors for prostate cancer have already been firmly identified. These include elder age, family history, race/ethnicity, geographic location, while other potential risk factors such as diet, obesity, insulin resistance and androgens levels have also been implicated [4]. Acquired or inherited genetic alterations in tumor suppressor genes, oncogenes or genes associated with angiogenesis and cell cycle also appear to be associated with the risk of developing prostate cancer and the aggressiveness of the tumor [3].

Among the numerous modifiable factors that have been implicated in the risk of development of prostate cancer, obesity is the one that has been the focus of the most intensive research given the fact that present knowledge does not allow definitive recommendations. The clarification of the underling mechanisms could lead to specific preventative interventions [4]. Obesity results from a complex interaction of genetic predisposition and the environment, with increased caloric intake and decreased physical activity, leading to chronic energy imbalance and expansion of dysfunctional adipose tissue mass [5–7]. Obesity has reached epidemic proportions, with more than one billion of adults being overweight, of which at least 300 million are obese worldwide, according to the World Health Organization (WHO) [8]. While it is estimated that these figures will continue to increase in the forth coming years [6,9].

Adipose tissue is a metabolically active organ involved in energy homeostasis, immunity and endocrine balance [10]. In obesity and in particular in visceral or abdominal obesity, the adipose tissue becomes dysfunctional contributing to the development of several pathologic conditions such as metabolic syndrome, type 2 diabetes, cardiovascular diseases and cancer [11]. Although still controversial, obesity has been associated with an increased risk of developing esophagus, colorectal cancer, breast and prostate cancer [9,10]. Obesity has also been associated with an increased risk of mortality from cancer of 52% and 88% in men and women, respectively [12,13]. In particular, obese men have an increased risk of disease progression after radical prostatectomy and are more likely to develop metastasis or die from prostate cancer compared to normal weight men. This supports the hypothesis that obesity is associated with a more aggressive prostate tumor biology [14]. Furthermore, a high density of peri-prostatic adipose tissue has also been associated with more aggressive forms of prostate tumors and poor prognosis [15,16]. These observations also suggest that obesity is more related to the aggressiveness of the tumors and not directly related to the risk of development of prostate cancer [17–19]. Tumor progression seems to depend not only on the tumor itself but also on the surrounding microenvironment [16,20]. Prostate tumor cells have been shown to be able to change their surrounding adipocyte phenotype by inducing loss of lipid content, increasing the metabolic activity, and altering the adipokines and adipocyte markers expression, which in turn may promote tumor cell growth [21].

Several mechanisms have been proposed to explain the association between obesity and prostate cancer. For example, the increased levels of estrogens, insulin and insulin-like growth factor 1 (IGF 1) [10,22], as well as changes in the immune response and adipokines expression [12,23], which lead to a pro-inflammatory state, triggering DNA damage and angiogenesis that could result in increased tumor cell survival, proliferation, invasion and metastasis [10].

Several adipokines, such as leptin, IGF 1, interleukin 6 (IL 6) and Vascular endothelial growth factor (VEGF), are increased in obesity [14]. These have been demonstrated to interfere with several signaling pathways and have mitogenic effects. Consequently, they have been implicated in the risk of development and progression of prostate cancer in the obese state [24,25].

The identification of the adiposity associated factors that could promote the proliferation and survival of prostate tumor cells is important to not allow the establishment of recommendations for preventive interventions and the development of targeted therapies for obesity associated prostate cancer. Therefore, the main goal of our study was to assess the effects of preadipocyte and adipocyte secreted factors in the proliferation, migration and invasiveness of prostate cancer cells, and the effects of the adiposity signals, insulin and leptin, in cell proliferation.

## Materials and Methods

### Cell cultures

RM1 androgen independent prostate carcinoma cells belong to a murine cell line (mouse prostate reconstitution, MPR)[26] kindly offered by Prof T. Thompson from MD Anderson Cancer Center, Houston, Texas, USA. The cell cultures were performed according to the protocol previously published and implemented by our research group [7]. Cancer cells were grown in Dulbecco's Modified Eagle Medium (DMEM) High Glucose (Sigma-Aldrich, St Louis, MO, USA) supplemented with 10% fetal bovine serum (FBS) (Gibco, Life Technologies, Grand Island, NY, USA), 1% Penicillin- streptomycin (Pen-Strep) (Sigma-Aldrich, USA) and 50mM Hepes buffer solution (Sigma-Aldrich, USA). Every time cells became 90% confluent, the cell culture was split by incubating the cells with 0.25% trypsin- Ethylenediaminetetraacetic acid (EDTA) solution (Sigma-Aldrich, USA). To determine the number of cells at each passage, the viable cells were counted using the Trypan Blue (Sigma-Aldrich, USA) exclusion assay on a Neubaeur Chamber. Passages 12, 13 and 14 (P12, 13, 14) were used for this study.

3T3-L1 preadipocyte cell line was purchased from a commercial supplier (Zen- Bio, Durham, NC, USA) (passage 14 and 15) was cultured in DMEM High Glucose (Sigma- Aldrich, USA) supplemented with 10% Newborn Calf Serum (NCS) (Sigma-Aldrich, USA), 1% Pen-Strep and 50mM Hepes buffer was used as basal medium. Cells were grown in 25cm^2^ flasks (TPP, Trasadingen, Switzerland) until reaching 70% confluence when they were passaged by incubating the cells with 0.25% trypsin-EDTA solution. Adipocyte differentiation was induced two days after the cells reached full confluence. For that, the culture medium were removed, cells were washed with 1% phosphate buffer saline (PBS) (Gibco, Life technologies, USA) and incubated with DMEM-F12 (Sigma-Aldrich, USA) supplemented with 10% fetal bovine and 1% Pen-Strep, and a differentiation cocktail containing 1 μM dexamethasone (Sigma-Aldrich, USA), 0.5 mM 3-Isobutyl-1-methylxanthine- IBMX (Sigma-Aldrich, USA), and 1 μg/ml human insulin (Novo Nordisk A/S, Bagsværd, Denmark). After 72h, the medium were replaced by DMEM-F12 supplemented as described above plus 1μg/ml insulin. Afterwards, cells were re-fed every 48h with DMEM-F12 supplemented with 10% fetal bovine serum and 1% Pen-Strep.

All cell cultures were handled in a laminar flow chamber and maintained at 37°C in an incubator (Heracell 150i, Thermo scientific, Waltham, MA USA) with 5% CO_2_.

### Determination of cell growth curves

RM1 cell proliferation was assessed using the indirect method for estimating cell number based on the mitochondrial dehydrogenase activity, which reduces sodium 2,3-bis[2-Methoxy-4-nitro-5-sulfophenyl]-2H-tetrazolium-5-carboxyanilide inner salt) (XTT) (Applichem, Darmstadt, Germany) dye to formazan.

Growth curves were performed using three different initial RM1 cell concentrations 2x10^4^ /mL, 5x10^4^/mL, 10x10^4/^mL or cell culture medium only used as blank control, in five replicates (5 wells) for each concentration seeded into 96 well plate (BD Falcon, Franklin Lakes, NJ, USA), in order to determine the optimal RM1 cell concentration to use in the subsequent studies. At each time point, 40 μL (20% culture media volume) of XTT reagent was added to each well and the absorbance was measured 12, 24, 36 and 48h in a plate reader at 450 and 620nm, at the several times points. The absorbance at 620 nm was used as a background measurement and was subtracted to the 450 nm absorbance value.

The cell proliferation was estimated by the percentage of XTT reduction calculated using the following equation:

$$\%Reduction\;=\;\left(A_{450}-A_{620}\right)\;/\;A_{B450}$$

### Cell proliferation assays

After determining the ideal initial RM1 cell concentration (5x10^4^ cell/mL) to be used in the subsequent studies, the effect of adipocyte and preadipocyte conditioned media in RM1 cell proliferation was evaluated.

To examine the effect of preadipocytes and adipocytes secretome on RM1 cell proliferation, preadipocytes serum free conditioned culture medium (PACM) was collected 24h after being in culture, while adipocytes serum free conditioned culture medium (ACM) was collected after 24h and 48h after being in culture. Both media were stored at -80°C until use. RM1 cells (5x10^4^ mL/well; P12, 13, 14) were seeded in five replicates into 96 well plates (BD Falcon) and incubated for 12 hours in complete media, after which the medium was removed and replaced by conditioned media (24h preadipocyte and 24h and 48h adipocyte conditioned media) for 24h. Preadipocyte and adipocyte basal serum free media (DMEM High glucose supplemented with 1% Pen-strep, 50mM Hepes Buffer and DMEM-F12 supplemented with 1% Pen-strep, respectively) were used as negative controls. After 24h, 40μL of XTT reagent was added to each well and the absorbance was measured as previously described.

To examine the effect of preadipocytes and adipocytes in co-culture with RM1 in prostate cancer cell proliferation, 24-well transwell culture system plate with 0.4 μm pore size Polyethylene terephthalate (PET) membrane (Corning incorporated Life Sciences, St. Lowell, MA,USA) were used. Preadipocytes and adipocytes were cultured in the bottom chamber, while RM1 cells (4x10^4^/well) were seeded in the upper chamber of the transwell system. To evaluated RM1 cell proliferation, 24 hours after incubation, cells were detached, suspended in 1% PBS/ 2% Bovine Serum Albumin (BSA) / 5mM EDTA buffer solution and counted in a Coulter EPICS XL flow cytometer (Beckman Coulter, Inc., Brea, CA, USA) using cell counting beads (Sigma-Aldrich, USA).

To evaluate the effect of insulin and leptin on the proliferation of RM1 cells the resazurin reduction method was used. For that RM1 cells were seeded (5x10^4^cel/mL) and cultured with different human insulin (0 nmol/L, 200nmol/L, 800nmol/L and 1600nmol/L) (Novo Nordisk A/S, Denmark) or leptin (0ng/mL, 10ng/mL, 50ng/mL and 100ng/mL) (H-5582, rec leptin, Bachem, Bubendorf, Switzerland) concentrations that were added to the culture media together with 100μL of resazurin reagent- alamar blue (AbD Serotec, Oxford, UK). Five replicates for each concentration and negative control containing medium culture only and resazurin reagent were used, and the absorbance was measured at each time point (0, 12, 24 and 36h) at 570 and 595 nm. The percentage of resazurin reduction was calculated using the following equation:

$$\%Reduction\;=\;((Eox_{\lambda }{}_{2}\;x\;A_{\lambda }{}_{1}-Eox_{\lambda }{}_{1}\;x\;A_{\lambda }{}_{2})/\;(Ered_{\lambda }{}_{1}\;x\;A_{blank}\;{{}_{\lambda }}_{2}-Ered_{\lambda }{}_{2}\;x\;A_{blank}\;{{}_{\lambda }}_{1}))\;x100$$

being λ2 = 595 nm, λ1 = 570 nm, Eox_570nm_ = 80,573, Eox_595nm_ = 117,216, Ered_570nm_ = 155,667 and Ered_595nm_ = 14,652.

### Migration assay

RM1 cells were seeded in 12 well plates and cultured in complete media until reaching 80% confluence. After that, a “wound” was produced in each well by scratching down the center of the well using a pipette tip, and the culture medium were replaced by preadipocyte and adipocyte conditioned media or complete media (negative control), using 3 replicates for each condition. Cellular migration was assessed by measuring the rate of “wound healing” by measuring the distance between the edges of the scratched region at 0h and 24h using image J software (originated at the National Institutes of Health, USA) in images captured at 100x using an Motic AE31 inverted microscopes (Motic, Hong Kong, China) with a digital camera (Motican 5.0 MP, Motic) and Motic Image Plus 2.0 ML software (Motic, Hong Kong, China).

### Cellular invasion assay

To evaluate the invasion capacity of RM1 cells, cell culture inserts with an 8.0μm pore size membrane (BD Biocoat 24-well Matrigel Chambers, BD Bioscience Bedford, MA, USA), were used according to the manufacturer’s protocol.

3T3-L1 cells were seeded in the bottom chamber of the well, cultivated and half of the cell cultures were differentiated into adipocytes according to the protocol previously described and after completion of adipocytes differentiation, the culture media of preadipocytes and adipocytes was replaced by serum free media. Three replicates have been used for each condition, cell free culture media was used as negative control and adipocyte basal media supplemented with 30% FBS was used as positive control.

Matrigel-coated inserts pre-incubated for 1 h with serum-free DMEM/DMEM-F12 were placed in the wells and RM1 cells (4x10^4^ cells/well) were seeded in the upper chamber and cultured in the same conditions of the bottom chamber. After 24 hours, the media was collected, the inserts were washed with PBS 1% and the cells were fixed in paraformaldehyde 4% for 15 min at room temperature. Cells on the top surface of the filters were wiped off with cotton swabs and the membrane was carefully cut and mounted in a slide with DAPI-Vectashield (Vector Laboratories, Burlingame, USA). For each membrane, ten images (200x) were captured using a fluorescence microscope (Olympus Bx 41, Olympus, Tokyo, Japan) with an Olympus DP 70 digital camera and DP controller 2.1.1.183 Olympus software (Olympus, Tokyo, Japan). The number of cell nuclei in each image was counted using the software Image J and the migration rate was estimated by taking into account the area of the field of view and the total area of the membrane.

For each assay to evaluate proliferation, migration and invasion, different controls have been used for preadipocytes and adipocytes, to minimize the interference of medium, since these cell cultures have different basal media.

### Statistical analysis

For each condition mean, standard error of the mean (SEM) and normality were calculated. To compare two independent experimental groups, T Student test or Mann-Whitney were used, considering whether the sample followed a normal distribution or not. For three or more independent groups with normal distribution, a simple analysis of variance (one way ANOVA) with post-hoc Newman Keuls test was used or an Anova Kruskal-Wallis with post-hoc Dunn's was performed when the sample failed to have a normal distribution.

A p<0.05 was considered statistically significant. All statistical analysis were performed using Excel and GraphPad Prism software (GraphPad Software, Inc., La Jola, CA) version 5 for Windows.

## Results

### RM1 cell proliferation

**Fig 1:**
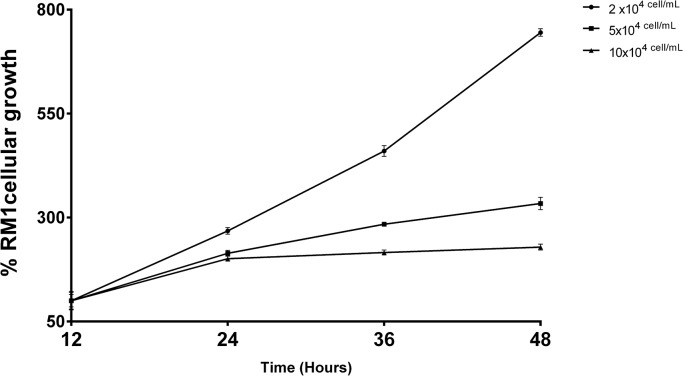
RM1 cell growth curve. Growth curve using three different initial cell concentrations (2x10^4^, 5x10^4^, 10x10^4^ cells/mL) evaluated by the determination of the percentage of tetrazolium salt reduction at different time points (12, 24, 36 and 48h).

The RM1 cell growth curve, performed by the XTT method, to choose the optimal cell concentration to be used in the studies to assess cell proliferation, has shown that the initial concentration of 5×10^4^cells/mL displayed a linear increase in the % of tetrazolium salt reduction, in the time interval to be used in most of the subsequent studies (Fig 1).

**Fig 2:**
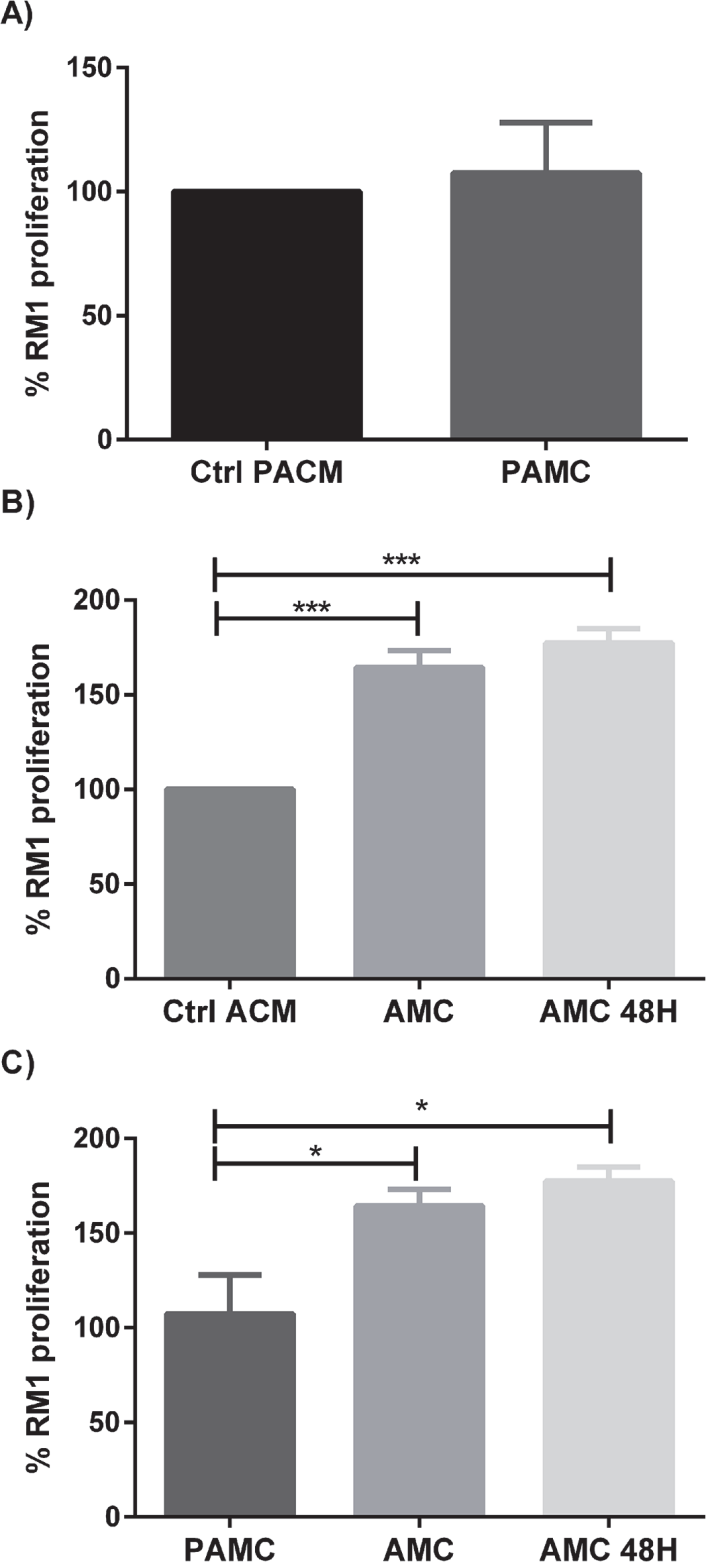
Cell proliferation assay in the presence of conditioned media. RM1 cell proliferation cultured with different conditioned media, assessed by the percentage of tetrazolium salt reduction. RM1 proliferation cultured with preadipocyte conditioned media (PACM) compared with the control (p>0.05, Mann-whitney U test) (A). RM1 proliferation cultured with 24 and 48h adipocyte conditioned media (ACM) compared with the control (*** p<0.001 one-way ANOVA, Newman-Keuls multiple comparison post-hoc test) (B). RM1 Proliferation with three different conditioned media (* p<0.05 one-way ANOVA, Newman-Keuls multiple comparison post-hoc test) (C).

The effects of preadipocyte conditioned media (PACM) and adipocyte conditioned media (ACM) (24h and 48h) on RM1 cell proliferation was measured by % XTT reduction. The adipocyte conditioned media increased significantly RM1 cell proliferation after 24h (p<0.001) when compared with controls (Fig 2A).Preadipocytes conditioned medium, despite triggering cellular proliferation compared to the control, failed to reach statistical significance (Fig 2B). The adipocyte conditioned media also increased significantly RM1 cell proliferation as compared with RM1 cells cultured with PACM (p<0.05) (Fig 2C).

**Fig 3:**
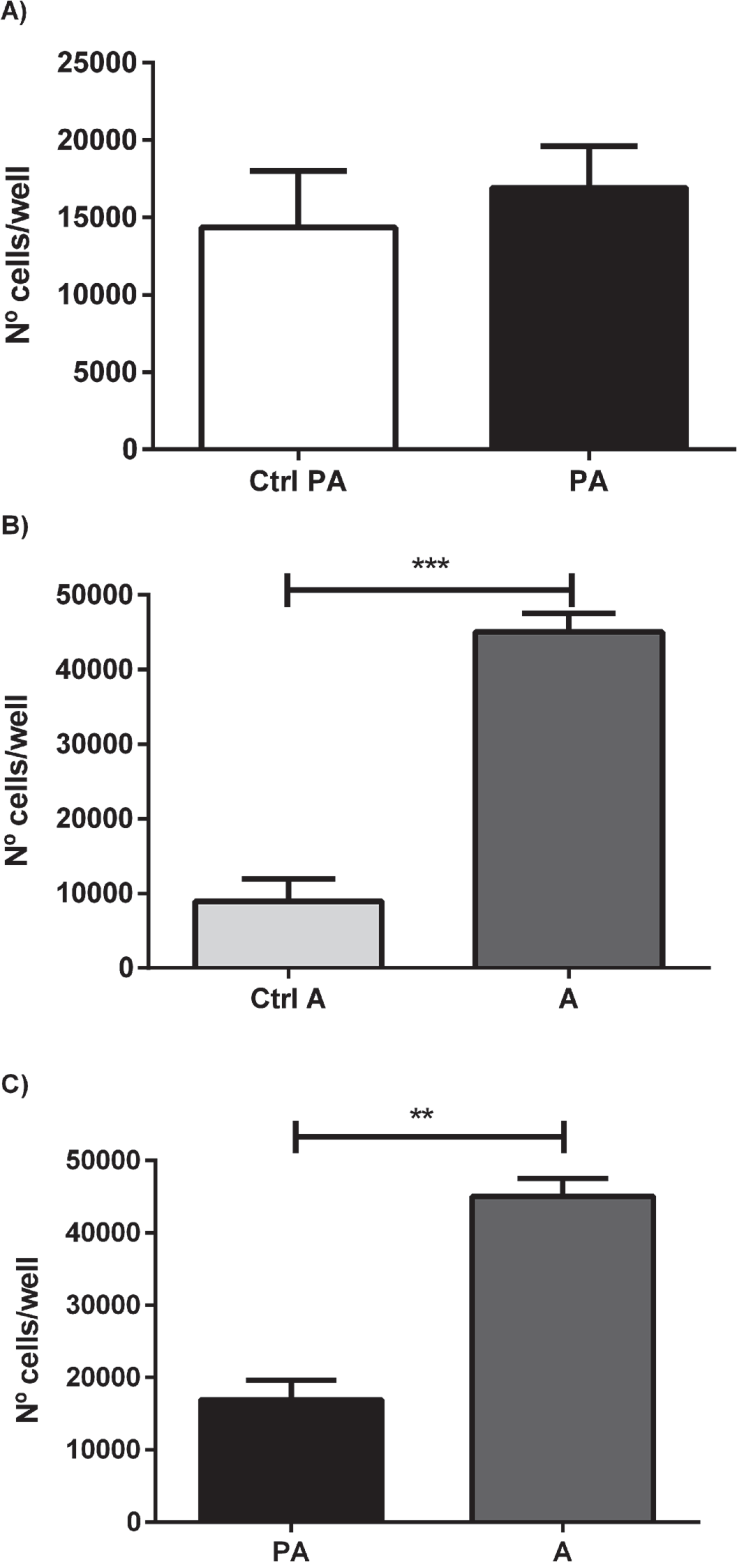
Cell proliferation assay in co-culture. Cellular proliferation was measured in RM1 co-cultivated with preadipocyte and adipocytes. Cell number/ well in different conditions tested, co-cultivated in the presence of preadipocytes (A) and adipocytes (B), all compared with the control, and comparing both conditions (C) (** p<0.01; *** p<0.001 Unpaired T test).

RM1 cell proliferation in co-culture with pre-adipocytes and adipocytes increased significantly in the presence of adipocytes (p <0.001), while co-culture with preadipocytes did not alter significantly RM1 cell proliferation when compared with the control (Fig 3A and 3B). The co-culture with adipocytes increased significantly RM1 cell proliferation compared with RM1 cells cultured with pre-adipocytes (p<0.01) (Fig 3C).

**Fig 4:**
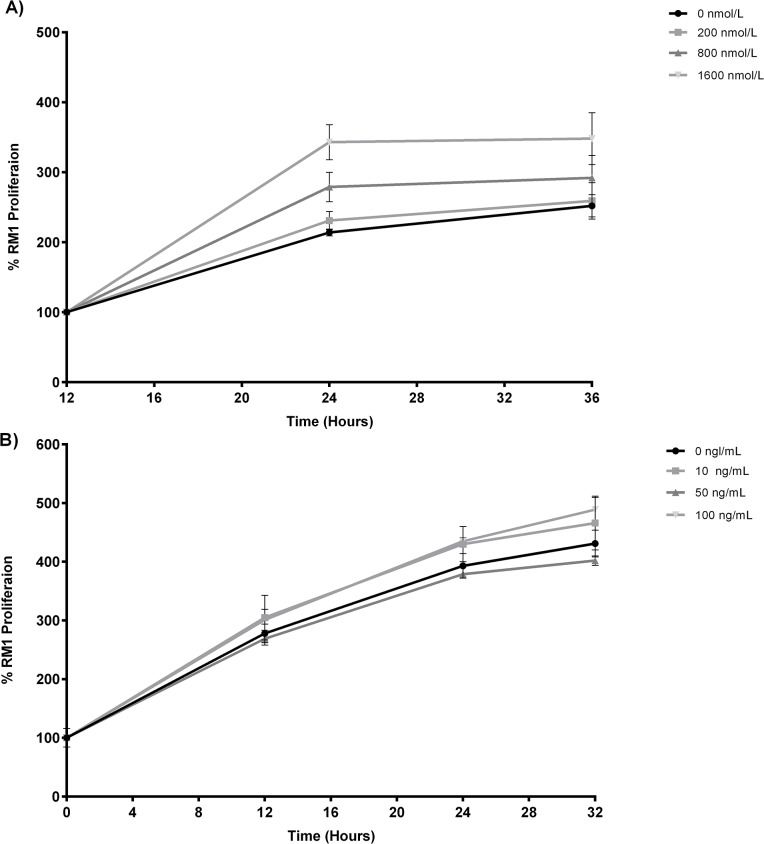
Cell proliferation assay in the presence of insulin e leptin. RM1 cell proliferation in the presence of different insulin concentrations (0, 200, 800, 1600 nmol/L) evaluated by determination of the % of resazurin reduction at different time points (A) (p<0.01 1600 nmol/L vs 800, 200, 0 nmol/L at 24h, one-way ANOVA, Newman-Keuls multiple comparison post-hoc test). RM1 cell proliferation of in the presence of different leptin concentrations (0, 10, 50, 100 ng/mL) evaluated by determination of the % of resazurin reduction at different time points (B).

RM1 cell (P12, 13 and 14) cultured in the presence of different insulin concentrations (0nmol/L, 200nmol/L, 800nmol/L and 1600nmol/L) increased significantly the percentage of resazurin reduction in the first 12 h after incubation, in dose dependent manner being highest at the higher insulin concentration (1600 nmol/L) (p<0.01). After 24h, there were no significant differences due to the larger variations (Fig 4A). Leptin produced no significant change in RM1 cell proliferation at the different concentrations tested (Fig 4B).

### RM1 cell migration capacity

**Fig 5:**
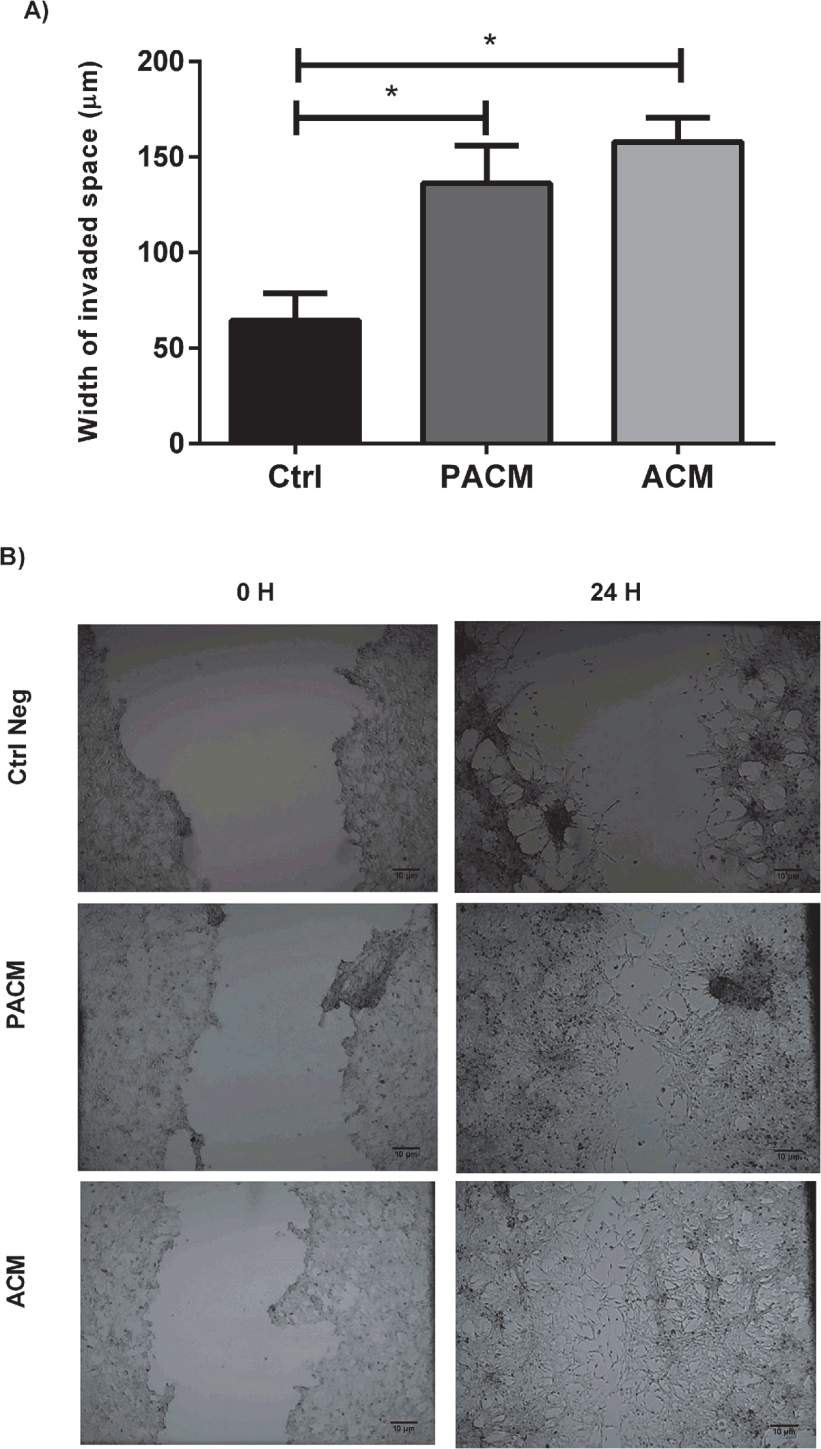
Migration assay. Width of invaded space by RM1 cells cultured with PACM, ACM and control media in the “wound healing assay” used to measure cell migration capacity (* p<0.05 one-way ANOVA, Newman-Keuls multiple comparison post-hoc test) (A). Photographs representative of the cellular migration in the “wound healing” process at the different experimental conditions measured at baseline (0H) and at 24H (B).

The migration capacity of RM1 cells assessed by the “wound healing” test has shown that cells cultured with adipocyte and preadipocyte conditioned media showed a significant increased migration capacity when compared with normal media (control) (p<0.05), being able to almost refill the wounded area after 24h (Fig 5A and 5B).

### RM1 cell invasion ability

The invasive potential of RM1 cells in the presence of preadipocytes and adipocytes was determined by the number of RM1 cells that invaded the Matrigel membrane, considering the microscope field area and the total area of the Matrigel membrane.

**Fig 6:**
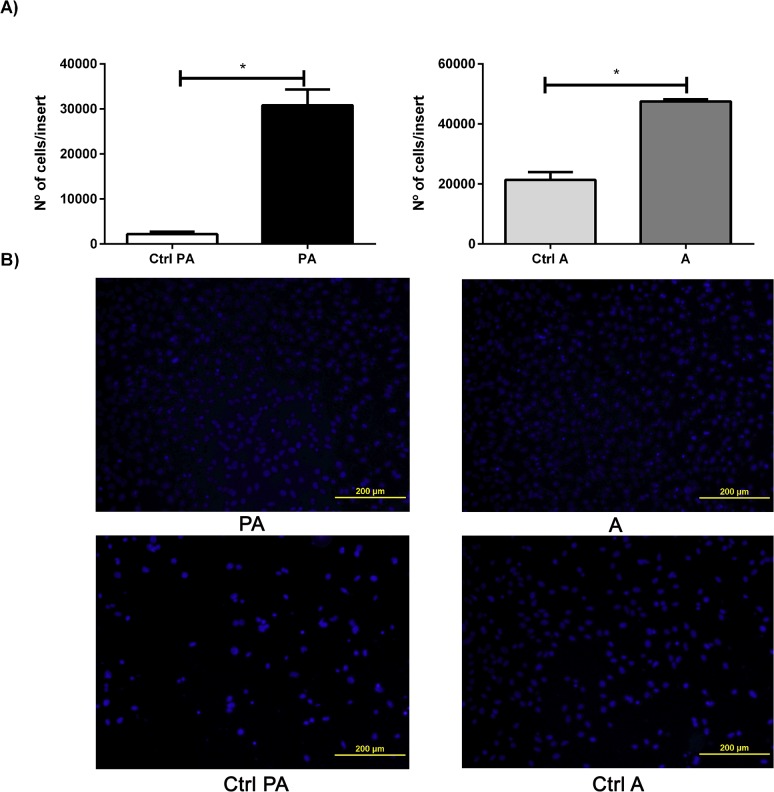
Invasion assay. Number of RM1 cells that invaded the matrigel membrane of the transwell system after 24 h in co-culture with preadipocytes, adipocytes and control media, used to evaluate invasion capacity, which was significantly higher in both co-cultures compared with control media alone (* p<0.05 Unpaired T test) (A). Representative images of the cells invading the matrigel membrane for each tested condition (B).

The number of RM1 cells that invaded the membrane was significantly increased in the presence of preadipocytes and adipocytes compared with the control (p <0.05) (Fig 6A and 6B). Comparing the effect of pre-adipocytes and adipocytes in RM1 cell invasion capacity, it was found that adipocytes where able to increase RM1 cells invasion ability comparatively to preadipocytes (Fig 6B).

## Discussion

Several epidemiological studies have suggested that there is an association among cancer incidence, mortality and obesity. Although controversial, this disease association varies considerably according to the tumor location. For example, in tumors located in organs surrounded by adipose tissue [11,27] the correlation is higher.

One of the suggested mechanisms for the obesity-associated risk of cancer is the chronic inflammatory state that characterizes obesity with increased expression of pro-inflammatory adipokines and species reactive oxygen (ROS) that could promote tumor progression, invasion and metastasis [11]. The purpose of this research work was to study the influence of adipocyte produced signals in the proliferation, invasion and migration of androgen independent prostate cancer cells.

The adipose tissue is known to participate in the metabolism of androgens, a well characterized stimulus for prostate cell growth, which could influence prostate cancer growth. Therefore, an androgen independent prostate cancer cell line was chosen as a model, in order to exclude the influence of sex steroids [28]. The role of adipokines in androgen-independent prostate cancer progression is also controversial and warrants further clarification if we consider that some authors argue that the effect of adipokines in tumor progression is higher in androgen-independent cells [14], while others claim that mechanisms other than adipokines should be the main proliferation triggers [11].

After selecting an initial cell concentration that displayed an exponential growth curve and without reaching the latent phase to evaluate the effect of preadipocyte and adipocyte secreted factors on prostate cancer cell proliferation, RM1 cells were cultured in the presence of different conditioned media collected from preadipocyte and adipocyte cultures. Adipocytes conditioned medium were obtained after differentiation of 3T3-L1 preadipocytes using a hormonal cocktail added to the culture medium capable of activating two transcription factors, CCAAT/Enhancer-Binding Protein β and δ. In turn, such factors stimulate the expression of adipocyte differentiation regulators, Peroxisome proliferator activated receptor γ (PPARγ) and CCAAT/enhancer binding protein α (C/EBPα), which induce adipogenesis transcription factors and expression of genes that determine the adipocyte characteristics [29,30]. Cell proliferation was assessed 24h later by the XTT indirect method that was used in alternative to the Alamar Blue method since the conditioned media was shown to interfere in the mechanism of the latter (personal data not shown). The preadipocyte conditioned media induced a non-significant increase in RM1 cell proliferation while adipocyte conditioned media caused a significant increase in proliferation when compared with control. This suggests that adipocyte secreted factors are able to interfere with prostate carcinoma cells proliferation as suggested by previous studies performed with DU145, PC3 and LNCaP prostate cancer cell lines [31]. It also corroborates the theory that proliferation and survival of prostate cancer cells are influenced by adipocytes [21,32]. Prostate cancer cell proliferation in co-culture with preadipocytes and adipocytes, so as to allow the diffusion of soluble factors and cell crosstalk but not direct heterotypic cell-cell contact, was found to be both significantly increased. Despite of this cell proliferation was significantly higher in cells co-cultured with adipocytes. These results are also in agreement with what has already been described by other authors who also found that the adipocytes where able to increase tumor cell proliferation [33–35] using prostate cancer cells and other types of tumor cells, in particular from colon and breast cancer.

Obesity has also been associated with the risk of prostate cancer progression and higher aggressiveness of the tumors. To test this hypothesis, the influence of adipocyte secreted factors in the capacity of migration and invasion of prostate cancer cells has been evaluated. Preadipocyte and adipocyte conditioned media significantly increased RM1 cell migration when compared with the control media results which are also consistent with previous migration studies performed using other prostate carcinoma cell lines. Such studies found that adipocyte conditioned media and sera from obese mice increased the migration rate of cancer cells cultured [14,36]. Our results further suggest that, although less prominent, preadipocytes also appear to be able to produce factors that can stimulate the motility and migration of prostate carcinoma cells, even though the specific signals, growth factors and cytokines involved in this mechanism are still to be identified.

Our studies have also shown that prostate cancer cells invasiveness capacity is significantly increased when cultured in the presence of preadipocytes and adipocytes. However, it is significantly higher in cells cultured in the presence of adipocytes. Thus, adipocyte secreted factors appear to be able to stimulate the migration and invasiveness of androgen-independent tumor cells, an effect that may be mediated by adipokines that either are not secreted or are secreted in smaller amounts by preadipocytes [34]. These effects had already been reported using other prostate carcinoma cell lines, as well as for other types of cancer cells, such as breast and ovarian cancer, which have also demonstrated to display an increased invasiveness capacity when cultured with adipocytes or their respective conditioned media [15,20,37,38]. Our results support the hypothesis that adipocyte secreted factors can enhance prostate cancer cell proliferation, invasion and migration and that these effects can be triggered not only by systemic mediators, such as insulin or cytokines secreted by the inflammatory cells in the adipose tissue, but also by specific and still unidentified adipocyte secreted molecules potentially produced by the interaction of tumor cells and with their microenvironment [14,33,35,38].

Although only leptin is secreted by the adipocyte cell, both, insulin and leptin, are the predominant adiposity signals in circulation [24,39], and therefore were chosen to assess the effects of obesity associated endocrine signals in prostate cancer cell proliferation. After having demonstrated in a previous study that RM1 prostate cancer cells express both leptin and insulin receptors [7], in the present study we show that high insulin concentrations (1600nmol / L) are able to trigger a significant increase in RM1 cell proliferation rate in the first 12h after incubation, when compared with lower concentrations (0 and 200 nmol/L) previously tested by us [40]. However, from 24h of insulin stimulation onwards, there no significant differences in proliferation were observed, most likely due to large variation observed. Since this variability was not observed in any of the previous studies in the absence of insulin, a possible explanation for the variation observed from 24h onwards after insulin stimulation is the heterogeneity of the cancer cell population, in result of the anaplastic nature of the cancer cell line with variable degrees of cellular differentiation, which might have resulted in different time lags in the response to the proliferation stimuli. These findings are in accordance with the results by Heidegger et al. that showed that insulin is able to stimulate LNCaP androgen-dependent and DU-145 and PC-3 androgen-independent prostate cancer cell proliferation [39]. Nonetheless, the same authors have also suggested that other signaling molecules such as IGF-1, may also be involved in the mechanism of insulin stimulated cell proliferation of prostate carcinoma [39,41], which binds to its own receptor Insulin like growth factor 1 receptor-IGFR but also to the insulin receptor (IR) and thereby activates two routes that promote proliferation [42]. Our results also support the hypothesis that high insulin concentrations might be able to activate mechanisms that increase cell survival and prostate cancer progression after androgens inactivation therapy or in castration-resistant forms [43].

In contrast, leptin in the different tested concentrations was not found to interfere significantly in prostate cancer cell proliferation. This is consistent with our previous results [40] in spite of the fact that other authors have demonstrated the pro-proliferative effects of leptin in androgen independent prostate carcinoma cells [24] and a risk association of high leptin levels in circulation with prostate cancer aggressiveness [44].

In summary, in the herein study, we demonstrate for the first time that adipocyte secreted factors are able to mediate pro-proliferative effects on prostate cancer cells, independently of mediators produced by other cell populations resident within the adipose tissue, such as inflammatory cells, which could also be responsible for driving these effects. Moreover, we have evaluated in the same experimental conditions the effects of adipocyte secreted factors on cell proliferation, migration and invasiveness, having demonstrated that these are able to mediate each of these phenomena. Since prostate cancer cells increased proliferation, migration and invasiveness, could all together contribute to enhance disease aggressiveness, this data thus supports the hypothesis that obesity can contribute to increase the risk of progression and aggressiveness of prostate cancer.
